# The complete mitochondrial genome sequence of white perch *Morone americana* (Perciformes, Moronidae)

**DOI:** 10.1080/23802359.2016.1172038

**Published:** 2016-06-20

**Authors:** Li Bian, Yongquan Su, Patrick M. Gaffney

**Affiliations:** a College of Ocean and Earth Sciences, Xiamen University, Xiamen, China;; b College of Earth, Ocean, and Environment, University of Delaware, Lewes, DE, USA

**Keywords:** Mitogenome, *Morone americana*, phylogenetic analysis, primer walking

## Abstract

The complete mitochondrial genome of the white perch *Morone americana* is described in this study. The total length of *M. americana* mitogenome is 17,966 bp, consisting of 13 protein-coding genes, 22 transfer RNAs, two ribosomal RNA genes and a noncoding control region. As with several other species in Moronidae, the ND6 gene in *M. americana* is found within the control region rather than at the canonical position between the ND5 and CYTB genes. Phylogenetic analysis based on CYTB gene places *M. americana* within Moronidae and confirms its close relationship with yellow perch (*M. mississippiensis*). This study provides a basic resource for further research on genetic structure and demographic history of *M. americana*.

The white perch *Morone americana* is an abundant semianadromous species found along the east coast of United States (Funderburk 1991). They are typically found in coastal bays and estuaries, as well as freshwater and brackish lakes and ponds (Woolcott 1962). Because of its abundance and euryhaline distribution in western Atlantic waters, *M. americana* is often used as an indicator species for contaminants (King et al. 2004; Hiramatsu et al. 2006; Barnthouse et al. 2009). To better understand spatial and ontogenetic patterns of contaminant accumulation in this species, genetic markers are needed to elucidate fine-scale population structure and demography. However, available molecular markers for *M. americana* are very limited at present. Therefore, we sequenced the complete mitochondrial genome of *M. americana*, to provide a valuable tool for continued investigation into population structure in this species.

Our sample was collected by bottom trawl in the upper Delaware Bay, USA in 2009. Total genomic DNA was extracted using a Qiagen DNA Isolation Kit (QIAGEN Inc., Valencia, CA). In order to sequence the mitochondrial genome, we first used several pairs of custom primers to amplify mitochondrial fragments containing ATP8, ATP6, ND2, ND4L and ND5. Then, we employed a primer-walking sequencing strategy to sequence the whole mitogenome. Polymerase chain reaction (PCR) primers and protocols used for this study are available upon request. Sequencher v.4.1.1 (Gene Codes Corp., Ann Arbor, MI) was used to make the assembly. Sequence chromatograms were examined in order to detect and correct sequencing errors. Three online programs – MitoAnnotator (Iwasaki et al. 2013), MITOS (Bernt et al. 2013) and DOGMA (Wyman et al. 2004) – were used to provide mitogenome annotations, As some annotations showed inconsistency, manual confirmation was performed by aligning the conflicting annotations to the mitogenome of three related species: *Morone saxatilis*, *Dicentrarchus labrax* and *Dicentrarchus punctatus* (GenBank accession number: HM447585, KJ168065, KJ168066).

In order to confirm the validity of the draft mitogenome, the complete CYTB nucleotide sequence of white perch and 17 other teleost species were used to construct a phylogenetic tree. A maximum-likelihood (ML) tree and a Bayesian tree were constructed. The ML tree was obtained using the online program PhyML 3.0 (Guindon et al. 2010) with 100 bootstrap replicates. MrBayes 3.2.5 (Ronquist et al. 2012) was used to build the Bayesian tree. The evolutionary model in MrBayes was set to the GTR substitution model with gamma-distributed rate variation across sites and a proportion of invariant sites. In order to clarify phylogenetic relationships within the genus *Morone*, four species (*M. americana*, *M. saxatilis*, *M. mississippiensis* and *M. chrysops*) were included to build another tree. DNA sequences from three mitochondrial genes (partial COXI, CYTB, ND6) of each species were concatenated and used to construct ML and Bayesian trees by as described previously.

The complete mitogenome sequence of *M. americana* contains 17,966 bp and has been deposited in GenBank with accession number KU641485. It consists of 13 protein-coding genes, 22 transfer RNAs, two ribosomal RNAs genes and a noncoding control region. Gene order is identical to the congeneric species *M. saxatilis*, except that annotated genome for the latter (HM447585) contains an additional tRNA-Ser. This inferred tRNA was not detected by MitoAnnotator, MITOS or tRNAScan-SE (Schattner et al. 2005), did not form a conventional tRNA-folding structure using RNAFold (Gruber et al. 2008) and had no match in the *M. americana* mitogenome. Therefore, it is unlikely to represent a real tRNA.

As with several other species in Moronidae (Williams et al. 2012; Tine et al. 2014), the ND6 gene in *M. americana* was found within the control region rather than the canonical position between the ND5 and CYTB genes. The other genes were found have a typical vertebrate mitochondrial gene arrangement. The whole base composition of *M. americana* was 29.1% C, 27.8% A, 26.9% T and 16.2% G. All the protein-coding genes began with ATG as the start codon, except for COXI, which started with GTG. For the stop codon, five genes used TAA, while the other eight genes had incomplete stop codons (TA or T), which were presumably completed as TAA by post-transcriptional polyadenylation (Anderson et al. 1981). Most of the genes were encoded on the H-strand, except for eight tRNA genes and ND6. The origin of *L*-strand replication (OL) in *M. americana* was found located between tRNA^Asn^ and tRNA^Cys^ genes within a cluster of five tRNA genes (tRNA-*Trp*, tRNA-*Ala*, tRNA-*Asn*, tRNA-*Cys* and tRNA-*Tyr*). Although the control region was separated by ND6 into two parts, we still identified one termination-associated sequence (TAS), one central conserved sequence block (CSB-D) and three conserved sequence blocks (CSB-1, CSB-2, CSB-3). We also found a 121bp tandem repeat sequence with eight complete repeats and one truncated repeat. In another *M. americana* control region sequence (HM447588) only four complete repeats and one truncated repeat were observed. Size heteroplasmy in the control region has also been documented in *M. saxatilis* (Chapman 1990; Wirgin et al. 1995; Williams et al. 2012) and *D. labrax* (Cesaroni et al. 1997).

The ML tree and Bayesian inference trees had the same topology (Figure 1), with the Perciformes and Anguilliformes (outgroup) falling into two clades. Within Perciformes, all the species under the same family were grouped together. The Moronidae species were placed as sister to the Lethrinidae. The Pomacanthidae and Chaetodontidae species were grouped as sister taxa. The four *Morone* and two *Dicentrarchus* species were clustered together with significant probability. Within Moronidae, the tree placed *M. americana* and *M. mississippiensis* as sister species and *D. labrax* sister to *D. punctatus*. The tree based on the concatenated nucleotide sequences of three mitochondrial genes showed similar results, with *M. americana* as sister taxon to *M. mississippiensis*, consistent with the conclusion of Leclerc et al. (1999) based on repetitive nuclear DNA sequences. The mitogenome of *M. americana* represents a useful resource for marker development for population studies, as well as for studies of phylogeny of Moronidae and related species.

**Figure 1.**
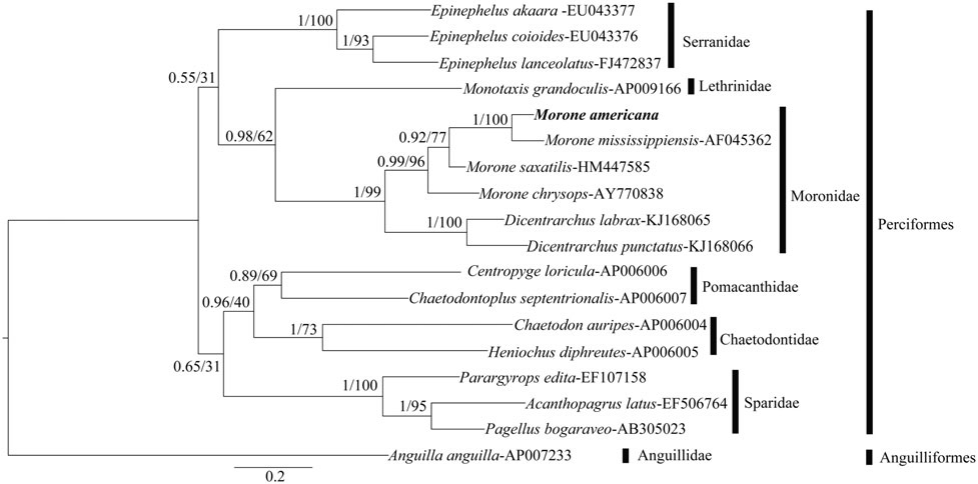
The Bayesian inference tree based on CYTB nucleotide sequence for 18 teleost species. For each node label, Bayesian posterior probabilities (left) and ML analysis bootstrap support value (right) are shown. Scale bar represents the expected substitution per site.
